# Prenatal diagnosis and prevention of toxoplasmosis in pregnant women in Northern Vietnam: study protocol

**DOI:** 10.1186/s12879-017-2446-1

**Published:** 2017-05-25

**Authors:** G. Suzanne A. Smit, Thi Lam Binh Vu, Trung Dung Do, Niko Speybroeck, Brecht Devleesschauwer, Elizaveta Padalko, Ellen Roets, Pierre Dorny

**Affiliations:** 1 Department of Virology, Parasitology and Immunology, Faculty of Veterinary Medicine, Ghent University, Salisburylaan 133, 9820 Merelbeke, Belgium; 2 Department of Biomedical Sciences, Institute of Tropical Medicine (ITM), Nationalestraat 155, B-2000 Antwerp, Belgium; 3 Institute of Health and Society (IRSS), Université catholique de Louvain, Clos Chapelle-aux-champs 30, 1200 Woluwe-Saint Lambert, Brussels, Belgium; 4 Parasitology Department of the National Institute of Malariology, Parasitology and Entomology (NIMPE), 245 Luong The Vinh, Nam Tu Liem, Ha Noi, Viet Nam; 5 Department of Public Health and Surveillance, Scientific Institute of Public Health (WIV-ISP), Juliette Wytsmanstraat 14, 1050 Brussels, Belgium; 6 Department of Clinical Biology, Microbiology and Immunology, Ghent University Hospital, De Pintelaan 185, 9000 Ghent, Belgium; 7 School of Life Sciences, Hasselt University, Agoralaan Building D, 3590 Diepenbeek, Belgium; 8 Women’s Clinic, Ghent University Hospital, De Pintelaan 185, 9000 Ghent, Belgium

**Keywords:** Congenital toxoplasmosis, Toxoplasmosis during pregnancy, Prenatal diagnostics, Serology, Prevention, Vietnam

## Abstract

**Background:**

In Vietnam, no systematic prenatal toxoplasmosis screening is in place, and only few studies have assessed the prevalence and importance of this zoonotic parasite infection. In addition, no studies have been conducted to assess the risk factors associated with toxoplasmosis. This study protocol was developed to determine the seroprevalence of toxoplasmosis in pregnant women in Hanoi and Thai Binh, Northern Vietnam, and to evaluate the association with risk factors and congenital toxoplasmosis. The protocol was developed in a way that it could potentially evolve into a countrywide prenatal diagnosis and prevention program, with the main focus on primary prevention.

**Methods:**

The collaborating gynaecologists will invite eligible pregnant women attending antenatal care for the first time to participate in the study. At first consult, information about toxoplasmosis and its prevention will be provided. All participants will be asked to fill in a questionnaire, which is designed to analyse socio-demographic and biologically plausible risk factors associated with toxoplasmosis, and blood samples will be collected to determine the seroprevalence of toxoplasmosis in pregnant women. In case there is suspicion of a primary infection during pregnancy, the concerned women will be followed-up by the gynaecologists according to a predefined protocol. Every participant will be informed on her serological status, risk factors and prevention measures and is offered appropriate medical information and medical follow-up if required.

**Discussion:**

The hypothesis is that congenital toxoplasmosis is an important but currently under-diagnosed public health problem in Vietnam. This study can strengthen sustainable control of toxoplasmosis in Vietnam, provide a protocol for prenatal diagnosis, boost overall awareness, improve the knowledge about toxoplasmosis prevention and can be essential for evidence-based health policy.

**Electronic supplementary material:**

The online version of this article (doi:10.1186/s12879-017-2446-1) contains supplementary material, which is available to authorized users.

## Background

Toxoplasmosis is a zoonosis caused by *Toxoplasma gondii*, a protozoan parasite, with felids as definitive hosts and warm-blooded animals as intermediate hosts. Humans get infected with *T. gondii* after consumption of raw or undercooked meat or ingestion of cat-shed oocysts via contaminated soil, food or water; or congenitally by transplacental transmission of tachyzoites [1, 2]. Congenital toxoplasmosis is asymptomatic in most cases but it can also result in fetal or neonatal death or various congenital defects, such as hydrocephalus, central nervous system abnormalities and chorioretinitis [3–5]. Acquired toxoplasmosis can cause serious disease in immunocompromised patients [6] but usually results in a relatively mild acute illness in immunocompetent individuals with some cases suffering fatigue and/or acquired chorioretinitis. However, there is increasing evidence that it may also result in a number of neurological or psychiatric diseases [7, 8].

The Foodborne Disease Burden Epidemiology Reference Group (FERG) ranked *T. gondii* as the 13th most important cause of foodborne diseases globally [9]. Congenital toxoplasmosis has an estimated global incidence of 1.5 (95% credibility interval (CI) 1.4–1.6) cases per 1000 live births, resulting in a burden of 9.6 (95% CI 5.8–15) Disability-Adjusted Life Years (DALYs) per 1000 live births [5]. The presence of cats, alimentary habits, hygienic conditions and the climate can cause the variation seen in prevalence in humans in different countries and within countries from one region to another [10–13].

Since congenital toxoplasmosis can only occur when a woman is primarily infected with *T. gondii* during pregnancy, it can be important to know her serological status at the beginning of pregnancy. Women who are seropositive have no risk for congenital toxoplasmosis and prevention measures can seriously reduce the risk of infection during pregnancy in seronegative women. Breugelmans et al. [14] concluded that the promotion of simple primary prevention measures is very effective in the prevention of toxoplasmosis during pregnancy. For these measures to be successful in the local context it is important to know the major risk factors associated with the infection.

Until now, few studies have been conducted on the prevalence and importance of toxoplasmosis in Vietnam, a densely populated country in southeast Asia with a population of around 93.5 million in 2015 [15]. Studies conducted between 1959 and 2003 showed a toxoplasmosis prevalence ranging from 7.7 to 29% in the general population and 11% in pregnant women [16, 17]. In a sero-survey on toxoplasmosis in rural areas of the northern provinces, Nghe An, Lao Cai and Tien Giang, a seroprevalence of 4.2% was found [18]. In animals, a seroprevalence of 27% was found in pigs [19] and of 11 and 3.0% in cattle and water buffaloes, respectively [20]. However, currently no systematic prenatal screening of toxoplasmosis is in place and no studies have assessed the risk factors associated with toxoplasmosis in Vietnam. Therefore, this study is aimed to determine the seroprevalence of toxoplasmosis in pregnant women in Hanoi and Thai Binh, Northern Vietnam, and to evaluate the association with risk factors and congenital toxoplasmosis. In addition, the protocol was developed in a way that it could potentially evolve into a countrywide prenatal diagnosis and prevention program, with the main focus on primary prevention.

## Methods

### Study sites and subject related procedures

The study will be conducted in collaboration with The Parasitology Department of the National Institute of Malariology, Parasitology and Entomology (NIMPE) in Hanoi, and the serological tests will be carried out in their laboratory. The two study sites are the National Hospital of Obstetrics and Gynaecology in Hanoi, one of the leading hospitals in Vietnam for obstetrics and gynaecology, and the Hospital of Obstetrics and Gynaecology in the Thai Binh province (120 km south east of Hanoi). In the first hospital approximately 200–300 pregnant women present for consultation and follow-up each day and in the second hospital around 150. Vietnam allows families to have a maximum of two children (decision no 162-HDBT, date 18 October 1988), which induced a decrease in the population size over the years. The country showed a crude birth rate of 16.2, with 15.3 in urban areas and 16.7 in rural areas in 2015. The age-specific fertility rate was 24 per 1000 women in the age category 15–19 years in 2009, compared to the highest rates of 121 and 133 per 1000 women in the age categories 20–24 and 25–29 years, respectively [15]. Hanoi, with geographical location (21°.2′N, 105°51′E), has a population size of 7.2 million and Thai Binh province, with geographical location (20° 30′N, 106° 20′E), has a population size of 1.8 million approximately [15]. In contrast to the rest of Vietnam, Thai Binh has a dense cat population, which consist of pets, street cats, and cats for meat consumption. Thai Binh is therefore also known as the “cat province” [21].

The cooperating gynaecologists will identify eligible pregnant women attending the antenatal care for the first time during the specified study period. In order to be eligible, study participants must meet the following criteria: pregnant woman; between 16 and 48 years old; gestational age ranging from 0 to 14 weeks; HIV negative; follow-up of pregnancy by one of the collaborating gynaecologists of the collaborating hospitals; willingness to participate during the total duration of pregnancy and if necessary after birth; willing and able to provide written informed consent (by signature of the patient or, in case the person is illiterate, by thumbprint and a signature of an impartial witness).

Both hospitals are public hospitals, accessible for women of all layers of the society. Women in Vietnam, especially in urban areas, tend to go the gynaecologist from the moment they suspect to be pregnant and go for follow up consultation and ultrasound every month until delivery. Since the study will only include pregnant women attending antenatal care for the first time it is unlikely we will have an overrepresentation of women with potential complications. Therefore, we believe that both the hospitals in Hanoi and Thai Binh can recruit a representative population of pregnant women.

Assuming an expected prevalence of 50% and an absolute precision of 5% at a 95% confidence level, the required sample size for estimating the seroprevalence in eligible pregnant women should be at least 385 per hospital [22]. To account for a small number of potential dropouts (which will be avoided as much as possible) we decided to increase the sample size to 400 per hospital.

At the first consult awareness will be raised on the importance of toxoplasmosis and its possible consequences on pregnancy. The benefits and risks for pregnant women to know their serological status will be explained and they will be asked by their gynaecologist to participate in the study and to fill in the informed consent form (see Additional file 1). In addition, they will receive an information folder in Vietnamese including prevention measures for toxoplasmosis, which will be reiterated mid-gestation (see Additional file 2). Participants will be asked to fill in a structured questionnaire to detect socio-demographic and biologically plausible risk factors associated with toxoplasmosis, awareness, clinical history and presentation of signs and symptoms relating to toxoplasmosis (see Additional file 3). During the same consult 5 ml blood will be collected from participating women by the gynaecologist in the hospital.

### Laboratory procedures

The blood will be collected in labelled tubes (with hospital code, identification code of participant, and sample number) and transferred in cool boxes on ice to the laboratory of the Parasitology Department, NIMPE, for testing within a week following sampling. Serum samples can be stored for up to 5 days at 2–8 °C; if longer storage is required, they can be frozen at −25 ± 6 °C. After separation of the serum by centrifugation, all samples will be tested for specific anti-*Toxoplasma* IgG by the Toxoscreen Direct Agglutination kit (BioMérieux, Marcy-l’Etoile, France) according to the manufacturers’ instructions. Screening of antibodies will be done on 1/40 and 1/4000 serum dilutions, the latter to monitor for a possible prozone effect at the 1/40 dilution.

To detect recent infections an IgM test will be conducted using the ISAGA kit (Immunoglobulin-M immunosorbent agglutination assay, BioMérieux, Marcy-l’Etoile, France), according to the manufacturers’ instructions. Detection of IgM and IgG is possible after approximately 2 and 4 weeks after infection, respectively*.* IgM is detectable by ISAGA for approximately a year (median 12.8 months, interquartile range 6.9–24.9) whereas IgG persists lifelong in immunocompetent people [23–25].

**Fig. 1:**
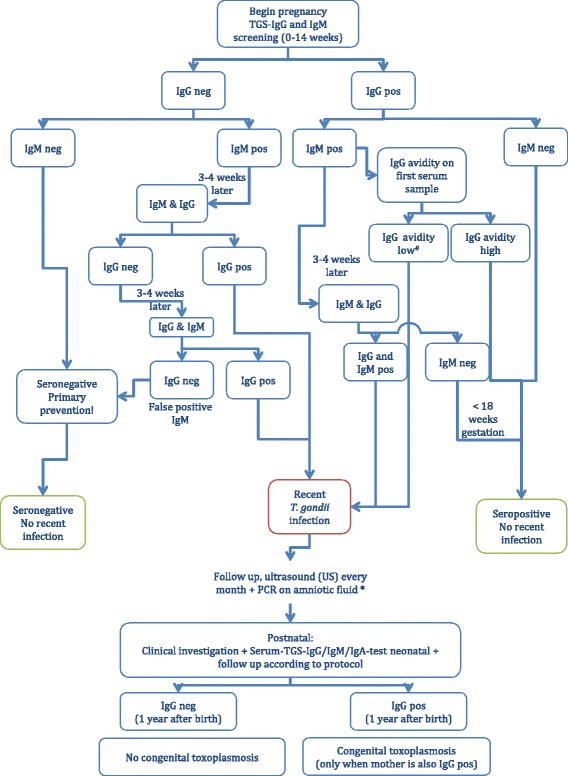
Diagnostic flowchart of congenital *Toxoplasma gondii* infection. Adapted from Van Haesebrouck et al. [28] and Montoya and Remington [27]. ^#^Low avidity can be an indication of recent infection but should not be directly interpreted as recent infection [26]. *Amniotic fluid PCR can be performed starting from 18 weeks of gestation onwards and at least 8 weeks after diagnosis of seroconversion. The risk of the procedure should be carefully weighed against the potential benefit of diagnosing fetal infection [27]. pos, positive; neg, negative; US, ultrasound; PCR, polymerase chain reaction; TGS, *Toxoplasma gondii* specific

Women that test positive for IgG only are considered seropositive and thus immune. Women who are IgM positive will be tested again for IgG and IgM 3–4 weeks later (Fig. 1). An IgG negative and IgM positive test result in the first test and an IgG positive test result 3–4 or 6–8 weeks later is indicative for seroconversion. When positive for IgG and IgM in the first test an IgG avidity test (on the first serum sample; performed in the National Hospital of Obstetrics and Gynaecology in Hanoi) will be done to determine the time of seroconversion.

The IgG avidity test measures the aggregate strength with which the antibodies bind to the antigens. The affinity increases over time of infection, which means high avidity indicates an old infection (>3 months ago). Low avidity can be an indication of recent infection, but some individuals have persistent low IgG avidity for many months after infection, which means it cannot be used alone to determine whether the infection was recently acquired [26].

At any suspicion of seroconversion, women will be followed-up (including an ultrasound every 4 weeks) by their treating gynaecologist. The gynaecologist will make sure these women will be advised correctly about the risks of transmission to the foetus, the risk of symptoms when there is a congenital infection, the steps that will have to be taken during follow-up, and all worries/anxieties/guilt will be carefully discussed and put into perspective. Amniotic fluid PCR can be performed starting from 18 weeks of gestation onwards and at least 8 weeks after diagnosis of seroconversion. The risk of the procedure should be carefully weighed against the potential benefit of diagnosing fetal infection [27].

Women that test negative for toxoplasmosis specific IgG and IgM in the first test will be informed by their gynaecologist about (congenital) toxoplasmosis as a disease and what measures should be taken to avoid toxoplasmosis during pregnancy. A folder (in Vietnamese) will be handed out to all participants explaining (congenital) toxoplasmosis and its prevention. These recommendations will be reiterated to seronegative women at around mid-gestation.

If there is any indication for congenital toxoplasmosis, NIMPE will make sure that the child will be correctly investigated and followed-up for any signs and symptoms of congenital toxoplasmosis by a neonatologist/paediatrician of the parents’ choice. For serology, blood samples will be collected by the treating neonatologist/paediatrician within 2 days and tested for toxoplasmosis specific IgM, IgA (performed in Belgium) and IgG. This will be repeated after 10 days and every month up to 4 months, and then at 6, 9 and 12 months after birth. NIMPE will take care of the follow-up serology and will give all necessary support to the treating paediatrician and parents. The presence of IgM and IgA in neonatal serum is diagnostic for congenital toxoplasmosis. However, the sensitivity is low and decreases when the infection occurred early during pregnancy. Persisting IgG antibodies 9 months to 1 year after birth are an indication for congenital toxoplasmosis but only if the mother is also found IgG positive in the perinatal period [28].

Table 1 shows an overview of the activities during the different antenatal consults. A detailed patient form should be filled in for every patient by the treating gynaecologist (see Additional file 4) and if follow-up and additional medical care is necessary, the gynaecologist can consult the provided doctors’ form with the most important information, including diagnostics, prognostics and options for treatment (available from the authors upon request).

**Table 1:** Overview of the activities during the different antenatal consults

	C1 Enrolment	C2 (3–4 weeks after C1)	C3 (6–8 weeks after C1)	C Mid gestation	N1 Postnatal
Eligibility screen	X				
Informed consent	X				
Questionnaire	X				
Information folder about toxoplasmosis and its prevention	X			X^c^	
Patient anamnesis	X				
Clinical examination	X	X	X	X	X
Blood sample for serology	X	X^a^	X^b^		
Blood sample from new-born for serology					X^d^

^a^If IgM positive; ^b^If IgG negative and IgM positive at C1 and C2; ^c^If seronegative; ^d^If suspicious for congenital toxoplasmosis. *C* prenatal consult, *N* neonatal consult

After finalization of each laboratory test, a picture will be made of the Toxoscreen-DA and ISAGA plates for documentation purposes and for interpretation support. All serum samples (frozen at −25 ± 6 °C) and documents will be retained at NIMPE for 5 years after the completion of the study, which assures the possibility to retrospectively look back at the results in case an asymptomatic child at birth shows any symptoms later in life.

The laboratory of the Parasitology Department, NIMPE, has received the ISO Certificate VILAS no 924 in May 2016. Local investigators will ensure compliance with protocols and quality of samples, documentation and analyzes. The results will always be validated according to the verification procedure set up in the lab for quality control.

### Data analysis

Data from the source documents will be entered in Microsoft Excel 2011 and access to the file will be protected by a password. Data and results will be handled confidentially and privacy of participants will be assured. All personal information is only documented in the file of the treating gynaecologist and will be encoded by a unique patient number. These codes will be linked to the respective informed consents, questionnaires, samples and patients’ forms thereby blinding investigators to direct identification of the data. The results of the blood testing will be communicated to the treating gynaecologists and from there to the participating women.

Cross-sectional studies are observational and descriptive studies and can be used to describe the seroprevalence and risk factors. In the group where there is suspicion of a seroconversion, the respective women will be followed-up (longitudinal) by their gynecologists.

The seroprevalence of toxoplasmosis in pregnant women in Hanoi and Thai Binh will be determined and a comparison will be made between these two regions. The overall incidence of toxoplasmosis can be calculated using a disease transmission model. The simplest disease transmission model is the so-called catalytic model, which divides the population into a seronegative and seropositive compartment. The rate at which seronegative individuals become positive over time is called the force of infection (λ), and is directly related to the overall incidence. Under the assumption of lifelong immunity and homogenous transmission dynamics over time, it can be shown that the age-specific seroprevalence π(a) is related to the age-specific force of infection λ(a). As a result, the force of infection can be statistically determined from the age-specific seroprevalence. The simplest model assumes an identical force of infection for each age group (i.e., λ(a) = λ) [29]. In reality, however, this assumption is not always accurate. Therefore, the force of infection can also be modelled as a linear or quadratic function, or, more flexibly, using fractional polynomials or splines [30]. We will choose the model that best suits the data.

Based on the incidence in the pregnant women, the incidence of congenital cases will be estimated. For toxoplasmosis it is commonly assumed that only primo-infection of seronegative mothers may lead to congenital infections [31]. The incidence of this congenital infection will depend on the force of infection, the age-specific seroprevalence of women, and the risk of trans-placental transfer of the pathogen. In addition, we will be able to assess the incidence of toxoplasmosis during the first trimester of pregnancy and the related incidence of congenital infections due to the follow-up of women that test positive for toxoplasmosis related IgM. Based on the information on the incidence in the first trimester, combined with information on the percentage of congenital infections seen in the first trimester [24] we can estimate the total incidence of congenital toxoplasmosis and this can be verified with the estimated incidence based on seroprevalence data. By combining the incidence estimate with information on the clinical impact from Torgerson and Mastroiacovo [5] and we will be able to estimate the public health impact of congenital toxoplasmosis in Northern Vietnam in terms of DALYs, which combine disease occurrence and clinical impact in a single number [32]. And by combining incidence estimates with the costs related to the protocol we will be able to assess the cost-effectiveness of this screening and prevention program.

The questionnaire will be analysed to detect socio-demographic and biologically plausible risk factors associated with toxoplasmosis, awareness, clinical history and presentation of signs and symptoms relating to toxoplasmosis. Multivariable analyses will be performed to investigate the association between *T. gondii* seropositivity as a dependent variable and possible demographic and risk factors as independent variables. To account for the study design, data analysis will be based on generalized linear models. All calculations will be performed in R 3.3.1 at the Institute of Tropical Medicine (ITM) in collaboration with NIMPE [33].

## Discussion

We hypothesise that the majority of the women of childbearing age will be seronegative in Vietnam, which makes dissemination of information about primary prevention an extremely important part of the study. We assume that the second largest group will be the seropositive women who are immune. A small group is assumed to have a seroconversion during pregnancy; this group requires appropriate medical care and follow-up.

Special care has to be given to women with a positive HIV status. In Vietnam, the prevalence of HIV is low. UNAIDS estimates of 2015 show a HIV prevalence rate of 0.5% [0.4–0.6%] in adults aged 15 to 49 years old [34]. Pregnant women are normally tested for HIV at the last antenatal consult before labour in our study sites. If the HIV status is already known at first antenatal consult, HIV positive women will be excluded from this study. However, prevention measures, which are of utmost importance in this group, will still be communicated. In addition, diagnostics for toxoplasmosis can still be carried out by NIMPE and correct medical follow-up and prophylaxis will be secured by the treating gynaecologist, according to the Vietnamese guidelines [35]. The neonatal ward should be notified about the use of trimethoprim-sulfamethoxazole or sulfadiazine in the mother near the time of delivery for early detection of hyperbilirubinaemia and kernicterus in the new-born.

If the HIV status was not known at first antenatal consult and the HIV test before labour turns out to be positive, this individual is excluded from the study. However, both a retrospective analysis of the toxoplasmosis serology and a new blood sample and diagnostics for toxoplasmosis will be carried out. Because negative *T. gondii* test results are seen in patients with abnormalities of B cell function, false negative results in HIV positive women are possible [36]. Pregnant women with primary toxoplasmosis have high risk of transmitting the infection to the fetus; the risk of transmission in reactivation of maternal infection due to HIV caused immunosuppression is lower [37]. The treating gynaecologist should perform ultrasound examination of pregnant women with HIV and toxoplasmosis for detection of hydrocephalus, cerebral calcifications, fetal growth restriction and other congenital toxoplasmosis related abnormalities. Furthermore, all children born to women with HIV need careful and thorough inspection and follow-up for any signs and symptoms of congenital toxoplasmosis and should be treated according to the diagnostic outcome by a pediatrician.

This project aims to strengthen sustainable control of toxoplasmosis in Vietnam. The hypothesis is that congenital toxoplasmosis is an important but currently under-diagnosed public health problem. Congenital toxoplasmosis not only potentially has a significant incidence in Vietnam but also induces long-term sequelae from an early age and thus has an important impact on the lives of patients and their families. We hypothesize that the seroprevalence is higher in Thai Binh compared to Hanoi due to the high cat population in the former. The seroconversion rate in pregnant women does not necessarily increase with a higher seroprevalence, since this depends on the age specific seroprevalence and incidence in women of childbearing age. A (single) serological screening for *T. gondii* antibodies can be recommended in women of child-bearing age as it allows identification of women at risk of acquiring infection and can be part of a strategic approach for the prevention of congenital toxoplasmosis [27, 38]. In addition, toxoplasmosis seroprevalence is continuously evolving, subject to regional socioeconomic parameters and population (health related) habits. Awareness of these trends, may guide the development of public health policies [39].

Environmental factors such as seasonal variation and variations due to rainfall might influence the force of infection [12, 40, 41]. However, we decided to focus our questionnaire on risk factors that can be influenced by awareness and implementation of prevention measures based on behavior. Our estimates will provide insights in the seroprevalence of toxoplasmosis in pregnant women and can provide a baseline against which future evolutions, e.g., associated with changing awareness, socioeconomic or environmental conditions, can be monitored.

This study will provide much needed information on *T. gondii* infection rates in pregnant women in Hanoi and Thai Binh. It will also establish a better understanding of toxoplasmosis epidemiology in pregnant women, thereby enabling a more structured and integrated control and prevention approach to reduce the risk of transmission and prevalence of this zoonosis. Furthermore, it will provide an assessment of the suitability and impact of toxoplasmosis screening and prevention information dissemination as an approach to the control of congenital toxoplasmosis, as well as a protocol that could potentially evolve into a countrywide prenatal diagnosis and prevention program, with the main focus on primary prevention.

Different screening strategies have been proposed in several settings differing in Toxoplasma epidemiological status, *T. gondii* strain and medical recourses, infrastructure and organization [38, 42–44]. This protocol can be used in similar settings and the choice of diagnostic test can be altered based on the resources, laboratory infrastructure, and experience. The ISAGA used in this study, ensures very early detection of IgM in a seroconversion and the test is very sensitive to residual IgM, while residual IgM can persist for periods of more than 1 year (according to the manufacturers’ instructions). Our protocol takes this into account by testing an IgG negative and IgM positive patient again 3–4 weeks later to identify IgG seroconversion and even a third time (6–8 weeks after the first test) if necessary. In case of residual IgM, our protocol describes to do an IgG avidity test to determine the time of seroconversion. The interpretation of the results should always be made taking into account the patient’s history and the results of all (other) tests performed. Since we acknowledge the diagnosis of toxoplasmosis can be complicated and may vary from person to person, our protocol allows retesting a sample with another test to compare outcomes if necessary and if there is any suspicion of seroconversion during pregnancy women will be thoroughly followed up (including an ultrasound every 4 weeks).

On the individual level, every participant will be informed on the test results and will be offered appropriate medical information and medical follow-up if required. In this way, every individual will know her serological status, risk factors and prevention measures. All participants in the study will be informed of the overall research findings once the project is completed.

In the long term, this study will contribute to increased knowledge and awareness of toxoplasmosis and its prevention and a reduction in the incidence of congenital toxoplasmosis, which can contribute towards sustainable development goal three by reducing child morbidity and mortality [45]. In addition, it is essential for evidence-based health policy, which can induce an important public health improvement and can reduce healthcare costs. Finally, information about the impact of this infection is of utmost importance for prioritizing the development of much needed prevention and intervention measures. Therefore, this project is a prerequisite for every further step forward.

## Additional files

Additional file 1: Informed consent form. (PDF 158 kb)

Additional file 2: Toxoplasma information folder. (PDF 952 kb)

Additional file 3: Questionnaire. (PDF 150 kb)

Additional file 4: Patients’ form. (PDF 713 kb)

## Abbreviations

DALY: Disability-Adjusted Life Year
ITM: Institute of Tropical Medicine
NIMPE: National Institute of Malariology, Parasitology and Entomology
