# Supplementary material for: Prenatal diagnosis and prevention of toxoplasmosis in pregnant women in Northern Vietnam: study protocol
Source: BMC Infect Dis. 2017 May 25;17:364. doi: 10.1186/s12879-017-2446-1 (PMC5445302; doi:10.1186/s12879-017-2446-1)
Supplement: Supplementary file 1 — Informed consent form. (PDF 158 kb) [file 12879_2017_2446_MOESM1_ESM.pdf]

Additional file 1

Informed consent form (women >16 years old)

Information

At the beginning (<14 weeks) of pregnancy during the first antenatal consult you are invited to take part in a research project on toxoplasmosis in Vietnam. The purpose of this form is to explain the study and ask for your consent (agreement) for your participation in this study. You are free to ask questions for clarification at all times.

The goal of this research is to estimate the presence of toxoplasmosis in pregnant women. Toxoplasmosis is a disease that results from infection with *Toxoplasma gondii*, one of the world's most common parasites. Only people, who never came in contact with the parasite before, can get infected. Humans may get infected by ingesting oocysts from the stool of cats via cleaning a cat litter box or by contaminated soil, food or water. Another route of infection with *T. gondii* can be through eating raw or undercooked meat. If women, who never came in contact with the parasite before, get infected during pregnancy the parasite can be transmitted from mother to child through the placenta. This is called a congenital infection or congenital toxoplasmosis. In most cases of congenital infection no disease is seen in the unborn child or the child after birth. However, some congenital infections may result in abortion/stillbirth or cause disease of the child, such as, abnormalities of the brain or the eyes. Some children do not show symptoms at birth, but can develop eye problems later in life. Toxoplasmosis can be prevented by reducing the risk of infection from food and the environment. You have received a detailed information form about toxoplasmosis and a list of prevention measures from your gynaecologist.

Next to studying the presence of toxoplasmosis, this research aims to evaluate the association between risk factors (such as cleaning the cat litter box or eating undercooked meat) and infection. In this way this study can help to find out the importance of toxoplasmosis and to make strategies to prevent congenital toxoplasmosis.

If you agree to participate in the study you will be followed up during the whole duration of your pregnancy. The further steps after signing the informed consent form will be filling in a questionnaire and giving a blood sample. Thereafter, the blood sample will be tested and you will be informed on the test results.
If the test is positive it means that you had the infection before pregnancy. This means you are immune so you cannot get infected again and the parasite cannot be transmitted to your child through the placenta. If the test is negative it means that you never came in contact with the parasite. This means you are not immune and it is possible to get the infection during pregnancy (or later in life). In this case, it is very important to follow the list of recommended prevention measures you received from your gynaecologist during pregnancy. If there is any suspicion for an infection during pregnancy you will be tested again and you will be offered appropriate support, medical information and medical follow up from your gynaecologist. If there is any indication for congenital toxoplasmosis (i.e. transmission of the infection to the unborn baby), your baby will undergo an appropriate investigation and follow-up for

any signs and symptoms of congenital toxoplasmosis by a paediatrician. Blood samples will be collected at specific time points (first sample within 2 days after birth) and will be tested for toxoplasmosis. This means your baby will get the appropriate follow up and treatment if required.

You will not be exposed to any risks. Only qualified health professionals will collect the blood samples to avoid discomfort. Because knowledge of the possibility of infection can cause stress, the treating gynaecologist will provide you with all the necessary support; follow up; information on toxoplasmosis and the ways to prevent this infection, to avoid stress as much as possible.

You will pay nothing and you will be compensated to participate in this study. You are free to refuse to participate and free to withdraw from the study at any time. Refusal of participation in the study will not affect the medical care you will receive.
All participants in the study will be informed of the research findings in general once the project is completed.

No participant will be identified by name, results will be handled confidentially and privacy of participants will be assured. Your personal information will be documented at your treating gynaecologist and will get a unique patient code. This code (not your personal information) shall be linked to the documents and blood sample, thereby blinding investigators to identification of the sample and information.

Collected blood samples may be exported for confirmation testing if necessary. Samples will be used for this study but will be stored at NIMPE for 5 years and may also be used for future studies (for example for other common pregnancy related tests) if you agree with this, the informed consent forms and questionnaires will be kept for 5 years. You have the right to see your data on demand at any time and to decide about such future use, to refuse storage and to have the material destroyed.

The study has been approved and will be monitored by the Ministry of Health, by the Institutional Review Board of the Institute of Tropical Medicine, Antwerp Belgium and the Ethical Committee of the Antwerp University Hospital (Belgium). This study is expected to benefit the community and generally contribute to knowledge on the disease.

Questions

For all questions and comments you can contact:

- Dr Vu Thi Lam Binh. National Institute of Malariology, Parasitology and Entomology (NIMPE), Parasitology Department, 245 Luong The Vinh, Tu Liem, Ha Noi, Viet Nam. Tel: +84 43 8542350. E-mail: lambinhdr@yahoo.com
- Institutional Review Board of the Institute of Tropical medicine, Nationalestraat 155, Antwerp, Belgium. Tel: +32.3.247.07.28. E-mail: acaron@itg.be

Patient code:

Written informed consent

I confirm that I have been informed about the study and that I have received a copy of the participant information sheet and the consent form. I have read and understood the information:

- I consent voluntarily to participate in this study
- I know that participation involves answering questions and blood sampling
- I know that participation might involve investigation, follow-up and blood sampling of my baby if there is any suspicion of congenital toxoplasmosis
- I am aware of the minimal risks involved from participating in this study (as explained above)
- I will have my follow-up of pregnancy by one of the collaborating gynaecologists of the collaborating hospitals
- I am willing to participate during the total duration of pregnancy
- I know that I can stop my participation in this study at any time
- I know that my personal information will not be used for identification of documents and samples because privacy will be ensured by coding all documents and blood samples with an unique patient code
- I know this data will be analysed and used only for research purposes and policy formulation
- I know that I have access to the data and records at any time

Do you consent that your blood sample may also be used for future studies (for example for other common pregnancy related tests)?
O Yes
O No

I have had all my questions answered to my satisfaction.
I have been asked to participate in the above research and given free consent by signing this form.

(a)……………………………………
Name of respondent

(b)…………………………………..
Signature or thumb print of respondent

(c)……………………………………
Signature of Researcher
Or Clinician

(d)……………………………………
Signature of witness

(e)……………………………………
Place

(f)……………………………………
Date
