# Supplementary material for: Prenatal diagnosis and prevention of toxoplasmosis in pregnant women in Northern Vietnam: study protocol
Source: BMC Infect Dis. 2017 May 25;17:364. doi: 10.1186/s12879-017-2446-1 (PMC5445302; doi:10.1186/s12879-017-2446-1)
Supplement: Supplementary file 2 — Toxoplasma information folder. (PDF 952 kb) [file 12879_2017_2446_MOESM2_ESM.pdf]

Toxoplasmosis Screening

What is toxoplasmosis?

Toxoplasmosis is a disease that results from infection with *Toxoplasma gondii*, one of the world's most common parasites. The parasite infects all mammals, including humans, and has the cat as definitive host. Only people, who never came in contact with the parasite before, can get infected. People who had the infection before are immune and are not at risk. Most people (90%) affected never develop signs and symptoms, but it may cause flu-like symptoms in some people, such as muscle aches and tender lymph nodes, and in a small number of people eye problems may develop. However, toxoplasmosis can cause complications in people with a weakened immune system or infants born to mothers, who never came in contact with the parasite before and get infected during pregnancy.

How do you get infected?

In the figure on the cover you can see the life cycle of the parasite. Humans may get infected by ingesting oocysts from the stool of cats via cleaning a cat litter box/ cat stool or by contaminated soil, food or water. Another route of infection with *T. gondii* can be through eating raw or undercooked meat.

What is congenital toxoplasmosis?

If women, who never came in contact with the parasite before, get infected during pregnancy the parasite can be transmitted from mother to child through the placenta. This is called a congenital infection or congenital toxoplasmosis. In most cases of congenital infection no disease is seen in the unborn child or the child after birth. However, some congenital infections may result in abortion/stillbirth or cause disease of the child, such as, abnormalities of the brain or the eyes. Some children do not show symptoms at birth, but can develop eye problems later in life. In the beginning of pregnancy the risk of transmission of the parasite from mother to child is small. However, the risk of symptoms in the child is larger than when transmission occurs at the end of pregnancy. Towards the end of pregnancy this risk of transmission of the parasite increases but the risk of symptoms in the child decreases.

How can congenital toxoplasmosis be prevented?

Reduce risk from food

- Cook food and especially meat well (well done – meat should not be "pink" in the center)
- Freeze meat for minimal 3 days in freezer (-20°C) before cooking
- Avoid smoked, cured, fermented, pickled or dried meats
- Avoid mucous membrane contact when handling raw meat
- Refrain from skinning and butchering animals
- Wash and peel fruits and vegetables thoroughly before eating
- Wash hands with soapy water after contact with raw meat, poultry, seafood, or unwashed fruits or vegetables
- Wash cutting boards, dishes, counters, utensils with hot soapy water after contact with raw meat, poultry, seafood, or unwashed fruits or vegetables preferably while wearing gloves or wash hands afterwards

Reduce risk from the environment

- Avoid drinking untreated drinking water
- Wear gloves when gardening and during any contact with soil, sand, floor, pavement or street because it might be contaminated with cat stool that contain *Toxoplasma gondii*. Wash hands with soap and warm water after gardening and/or contact with soil, sand, floor, pavement or the street
- Teach children the importance of washing hands to prevent infection
- Keep outdoor sandboxes covered
- Feed cats only canned or dried commercial food or well-cooked table food, not raw or undercooked meats
- Change the litter box/ clean up cat feaces daily if you own a cat. The *Toxoplasma gondii* parasite does not become infectious until 1 to 5 days after it is shed in a cat's stool. If you are pregnant or immunocompromised:
  - Avoid changing cat litter/ cat feaces if possible. If no one else can perform the task, wear disposable gloves and wash your hands with soap and warm water afterwards
  - Keep cats indoors
  - Do not adopt or handle stray cats, especially kittens. Do not get a new cat while you are pregnant

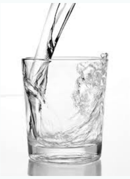

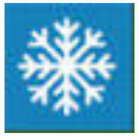

Adapted from: https://www.cdc.gov/parasites/toxoplasmosis/prevent.html

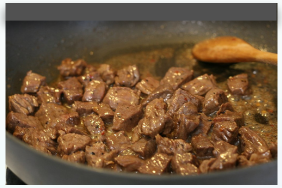

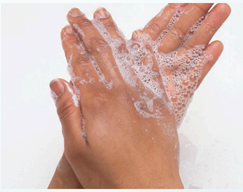

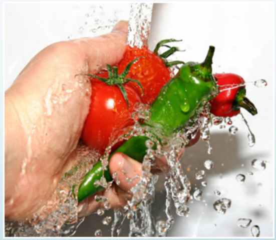

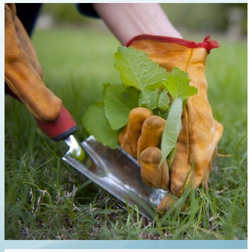

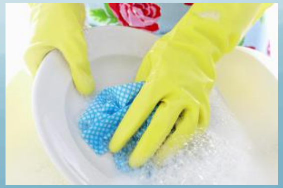

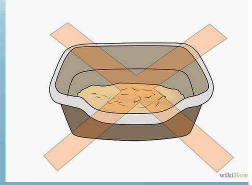

Why this study?

Testing pregnant women for an infection with toxoplasma allows identification of women at risk of acquiring infection. It is important that especially this group applies all the preventive measures. However, in Vietnam currently no systematic screening is conducted. Only few studies have been done on the presence and importance of toxoplasmosis in Vietnam. Furthermore, no studies have been conducted to assess the risk factors for toxoplasmosis. The hypothesis is that congenital toxoplasmosis is an important and currently underestimated public health problem in Vietnam.

The goal of this research is to estimate the presence of toxoplasmosis in pregnant women. In addition, it aims to evaluate the association between risk factors and infection. In this way this study can help to find out the importance of toxoplasmosis and to develop strategies to prevent congenital toxoplasmosis in the future.

Questions

For all questions and comments concerning the study you can contact:

Dr Vu Thi Lam Binh

National Institute of Malariology, Parasitology and Entomology (NIMPE)

Parasitology Department

245 Luong The Vinh, Tu Liem, Ha Noi, Viet Nam

+84 43 8542350

lambinhdr@yahoo.com

Toxoplasmosis Screening in Vietnam

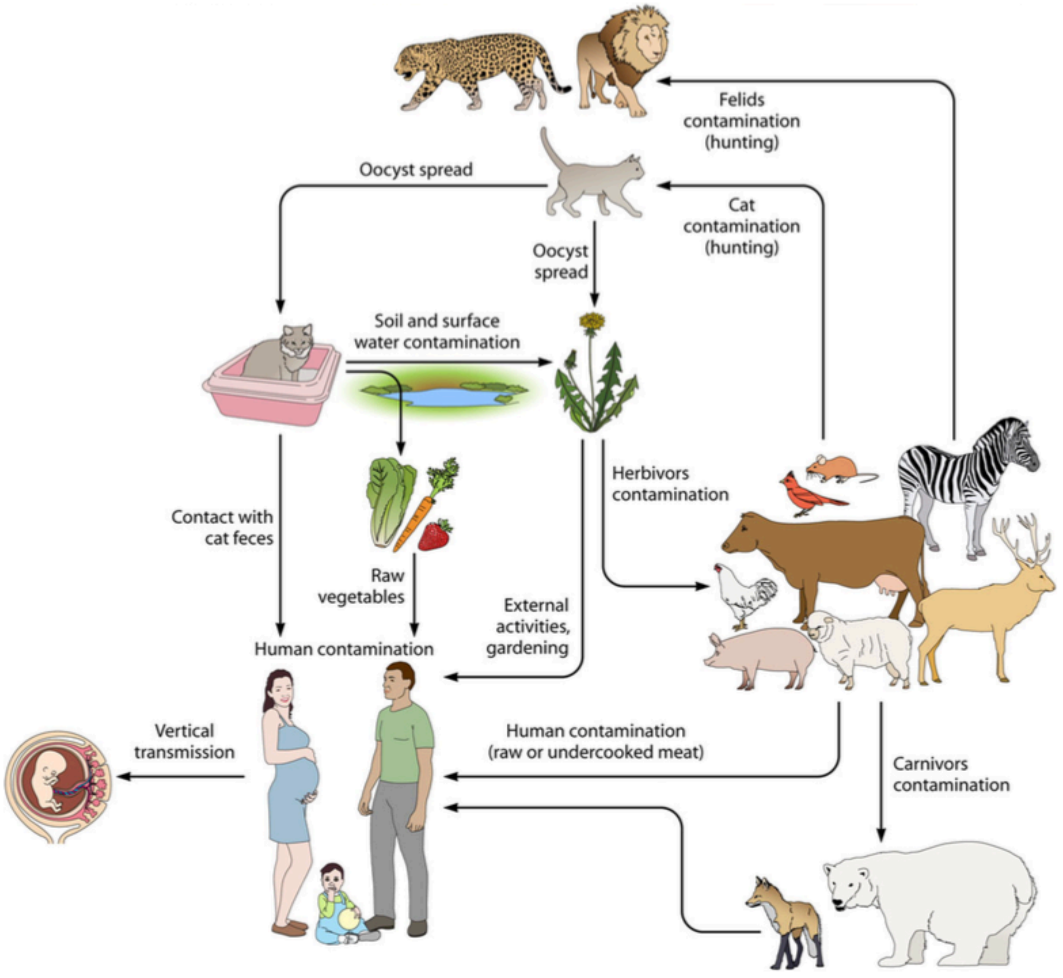

From: Robert-Gangneux F, Dardé M. Epidemiology of and Diagnostic Strategies for Toxoplasmosis. Clin Microbiol Rev. 2012; 25(2):264
