# Supplementary material for: Prenatal diagnosis and prevention of toxoplasmosis in pregnant women in Northern Vietnam: study protocol
Source: BMC Infect Dis. 2017 May 25;17:364. doi: 10.1186/s12879-017-2446-1 (PMC5445302; doi:10.1186/s12879-017-2446-1)
Supplement: Supplementary file 4 — Patients’ form. (PDF 713 kb) [file 12879_2017_2446_MOESM4_ESM.pdf]

Additional file 4

Patients' form *(Keep this page in the hospital – private information)*

Name doctor: ____

Hospital: ____

Date: ________________

Section I: Patient identification

Hospital code: [ ]

Patient label: [ ]

Name patient: ____

Age: ____

Address: ____

Zip code: ____

Place of residence: ____

Email address: ____

Telephone number: ____

Telephone number contact person: ________________________________

Comments:

____

____

____

Patients' form *(send a copy of the respective page(s) to NIMPE after every consultation together with the blood sample if applicable)*

Checklist 1st consult (C1)

Hospital code: [ ]

Patient label: [ ]

Date: [/ /]

**Activities 1st consult**	**Performed Yes/No** *(circle the applicable answer)*	**Comment**
Informed Consent	Y / N: why not?	
Questionnaire	Y / N: why not?	
Information and prevention leaflet about toxoplasmosis	Y / N: why not?	
Patient anamnesis	Y / N	
Clinical examination	Y / N	
Ultrasound	Y / N	
Blood sample for serology	Y / N: why not?	
Blood sent to NIMPE	Y / N: why not?	

Patient anamnesis

1. Age: ____
2. Place of residence/ Zip code: ______________________________
3. Gestational weeks: _____________________________________
4. Number of previous pregnancies: ___________________________
5. Previous stillbirths: No ☐ Yes ☐ please specify ___________________

6. Did the patient have any of the following conditions during the current pregnancy? (*Checking more than one checkbox is possible*)
   - ◻ Mononucleosis like symptoms *(e.g. bilateral, non-tender cervical or axillary lymphadenopathy, flu-like syndrome like fever, malaise, myalgia, hepatosplenomegaly, and pharyngitis)*
     Please specify:
     ____
   - ◻ Chorioretinitis *(e.g. ocular pain, blurred vision, blindness)*
     Please specify:
     ____
   - ◻ Central nervous system abnormalities *(e.g. headache, altered mental status, seizures, coma, fever, focal neurologic deficits, such as motor or sensory loss, cranial nerve palsies, visual abnormalities, and focal seizures)*
     Please specify:
     ____
   - ◻ None of the above
7. Did the patient have any other conditions during the current pregnancy?
   - ◻ No
   - ◻ Yes - Please Specify:
     ____
8. Did the patient have any lab results during the current pregnancy?
   - ◻ No
   - ◻ Yes - Please Specify:
     ____

Clinical information 1st consult (C1)

➢ Clinical examination results1st consult:
- ◻ Normal
- ◻ Abnormal - Please Specify:

____

____

➢ Ultrasound result 1st consult:
- ◻ Normal
- ◻ Abnormal - Please Specify:

____

____

Outcome 1st consult (C1)

Patient label:

Date: / /

Outcome 1st consult	Pos/Neg or Y/N *(circle the applicable answer)*		Comment
IgG	Positive(+)	Negative(-)	Titer:
IgM	Positive(+)	Negative(-)	ISAGA index:
Serology result communicated to the patient?	Y / N	Why not?	
Recommendation for follow up communicated to the patient?	Y / N	◻ IgG - & IgM -	Seronegative - Primary prevention very important!
		◻ IgG + & IgM +	Suspicious – Avidity test will be conducted and outcome will be communicated to you by NIMPE<br>Follow-up:<br>Take 2nd blood sample 3-4 weeks later (at 2nd consult C2)
		◻ IgG - & IgM +	Suspicious – Follow-up<br>Take 2nd blood sample 3-4 weeks later (at 2nd consult C2)
		◻ IgG + & IgM -	If < 18 weeks gestation,<br>Seropositive – Immune<br>No follow-up needed
		◻ Other - please specify:	

➢ Other Comments 1st consult:

Patients' form *(send a copy of the respective page(s) to NIMPE after every consultation together with the blood sample if applicable)*

Checklist 2nd consult (C2) – 3 to 4 weeks after first consult

Patient label: [ ]

Date: [/ /]

Checklist 2nd consult (C2) – 3 to 4 weeks after first consult

**Activities 2nd consult**	**Performed Yes/No** *(circle the applicable answer)*	**Comment**
Clinical examination	Y / N	
Ultrasound	Y / N	
Blood sample for serology if IgM positive at first consult	Y / N: why not?	
Blood sent to NIMPE	Y / N: why not?	

Clinical information 2nd consult (C2) – 3 to 4 weeks after first consult

- Clinical examination results 2nd consult:
  - ☐ Normal
  - ☐ Abnormal - Please Specify:

    ____

    ____

    ____

- Ultrasound result 2nd consult:
  - ☐ Normal
  - ☐ Abnormal - Please Specify:

    ____

    ____

    ____

Outcome 2nd consult (C2) – 3 to 4 weeks after first consult

Date: / /

Outcome 2nd consult	Pos/Neg or Y/N *(circle the applicable answer)*		Comment
IgG	Positive(+)	Negative(-)	Titer:
IgM	Positive(+)	Negative(-)	ISAGA index:
Serology result communicated to the patient?	Y / N	Why not?	
Recommendation for follow up communicated to the patient?	Y / N	**If 1st blood sample result was IgG + & IgM + And the 2nd sample result is the following:**	
		□ IgG + & IgM -	If < 18 weeks gestation and high avidity: Seropositive – Immune No follow-up needed
		□ IgG + & IgM +	Suspicious – Depending on the outcome of the avidity test thorough follow-up including ultrasound and if desired amniocentesis (>18 weeks of gestation and >8 weeks after seroconversion)
		□ Other - please specify:	
		**If 1st blood sample result was IgG – & IgM + And the 2nd sample result is the following:**	
		□ IgG - & IgM -	Seronegative - Primary prevention very important!
		□ IgG - & IgM +	Suspicious – Take 3rd blood sample 3-4 weeks later
		□ IgG + & IgM -	Seroconversion – Thorough follow-up including ultrasound and if desired amniocentesis (>18 weeks of gestation and >8 weeks after seroconversion)
		□ IgG + & IgM +	Seroconversion – Thorough follow-up including ultrasound and if desired amniocentesis (>18 weeks of gestation and >8 weeks after seroconversion)
Other laboratory results?	Y / N	Please specify:	
Treatment?	Y / N	Please specify:	

- Other Comments 2nd consult:

Patients' form *(send a copy of the respective page(s) to NIMPE after every consultation together with the blood sample if applicable)*

Checklist 3rd consult (C3) – 6 to 8 weeks after first consult

Patient label:

Date: / /

Checklist 3rd consult (C3) – 6 to 8 weeks after first consult

**Activities 3rd consult**	**Performed Yes/No** *(circle the applicable answer)*	**Comment**
Clinical examination	Y / N	
Ultrasound	Y / N	
Blood sample for serology if IgG negative & IgM positive at first and second consult	Y / N: why not?	
Blood sent to NIMPE	Y / N: why not?	

Clinical information 3rd consult (C3) – 6 to 8 weeks after first consult

- Clinical examination results 3rd consult:
  - ☐ Normal
  - ☐ Abnormal - Please Specify:

  ____

  ____

  ____

- Ultrasound result 3rd consult:
  - ☐ Normal
  - ☐ Abnormal - Please Specify:

  ____

  ____

  ____

Outcome 3rd consult (C3) – 6 to 8 weeks after first consult

Date: / /

**Outcome 3rd consult**	**Pos/Neg or Y/N** *(circle the applicable answer)*		**Comment**	
IgG	Positive(+)	Negative(-)	Titer:	
IgM	Positive(+)	Negative(-)	ISAGA index:	
Serology result communicated to the patient?	Y / N	Why not?		
Recommendation for follow up communicated to the patient?	Y / N	**If 2nd blood sample result was IgG – & IgM + And the 3rd sample result is the following:**		
		□	IgG - & IgM -	Seronegative - Primary prevention very important!
		□	IgG - & IgM +	Seronegative - Primary prevention very important!
		□	IgG + & IgM -	Seroconversion – Thorough follow-up including ultrasound and if desired amniocentesis (>18 weeks of gestation and >8 weeks after seroconversion)
		□	IgG + & IgM +	Seroconversion – Thorough follow-up including ultrasound and if desired amniocentesis (>18 weeks of gestation gestation and >8 weeks after seroconversion)
Other laboratory results?	Y / N	□		
Treatment?	Y / N	□		

- Other Comments 3rd consult:

___

___

___

Patients' form *(send a copy of the respective page(s) to NIMPE after every consultation together with the blood sample if applicable)*

Checklist consult mid gestation (Vmid)

Patient label:

Date: / /

Checklist consult mid gestation (Vmid)

Activities mid gestation	Yes/No *(circle the applicable answer)*	Comment
Did you give the information and prevention leaflet again to women who were seronegative	Y / N: why not?	

Patients' form *(send the respective page(s) to NIMPE after every consultation together with the blood sample if applicable)*

Checklist consult post natal (N1) – Only in case of suspicion of primary infection during pregnancy

Name doctor: [ ]

____

Hospital: ____

Date: ________________

Section I: Patient identification

Hospital code: [ ]

Patient label: [ ]

Name Mother: ____

Age: ____

Name Newborn: ____

Address: ____

Zip code: ____

Place of residence: ____

Email address: ____

Telephone number: ____

Telephone number contact person: ______________________________

Comments:

____

____

____

Date: / /

Checklist consult post natal (N1)

Activities post natal	Yes/No *(circle the applicable answer)*	Comment
Clinical examination mother	Y / N	
Clinical examination newborn	Y / N	
Blood sample from newborn for serology	Y / N: why not?	
Blood sent to NIMPE	Y / N: why not?	
Routine laboratory diagnostics including haematology and liver functioning tests	Y / N: why not?	
Ultrasound/MRI/CT brain	Y / N: why not?	
Eyefundus	Y / N: why not?	
Hearing screening	Y / N: why not?	

Clinical information consult post natal (N1)

- Clinical examination results **mother** post natal:
  - ☐ Normal
  - ☐ Abnormal - Please Specify:

  ____

  ____

- Clinical examination results **newborn** post natal:
  - ☐ Normal
  - ☐ Abnormal - Please Specify:

  ____

  ____

Outcome consult post natal (N1)

Patient label:

Date: / /

Outcome 2nd consult	Pos/Neg or Y/N *(circle the applicable answer)*		Comment
IgG	Positive(+)	Negative(-)	
IgM	Positive(+)	Negative(-)	
IgA	Positive(+)	Negative(-)	
Serology result communicated to the mother?	Y / N	Why not?	
Recommendation for follow up communicated to the patient?	Y / N	**See Protocol**	
Treatment initiated?	Y / N	Please specify:	

- Result of the routine laboratory diagnostics including haematology and liver functioning tests
  - □ Normal
  - □ Abnormal - Please Specify all abnormal test results:

____

____

____

____

- Result of the ultrasound/MRI/CT brain of the newborn
  - □ Normal
  - □ Abnormal - Please Specify all abnormal test results:

____

____

____

____

- Result of the eyefundus
  - ☐ Normal
  - ☐ Abnormal - Please Specify all abnormal test results:

____

____

____

____

- Result of the hearing screening
  - ☐ Normal
  - ☐ Abnormal - Please Specify all abnormal test results:

____

____

____

____

End

This is the end of the patient form. Thank you very much for your cooperation

For all questions and comments you can contact:

Dr Vu Thi Lam Binh. National Institute of Malariology, Parasitology and Entomology (NIMPE), Parasitology Department, 245 Luong The Vinh, Tu Liem, Ha Noi, Viet Nam. Tel: +84 43 8542350. E-mail: lambinhdr@yahoo.com
